# Organoid-based expansion of patient-derived primary alveolar type 2 cells for establishment of alveolus epithelial Lung-Chip cultures

**DOI:** 10.1152/ajplung.00153.2021

**Published:** 2022-02-09

**Authors:** Sander van Riet, Annemarie van Schadewijk, P. Padmini S. J. Khedoe, Ronald W. A. L. Limpens, Montserrat Bárcena, Jan Stolk, Pieter S. Hiemstra, Anne M. van der Does

**Affiliations:** ^1^Department of Pulmonology, Leiden University Medical Center, Leiden, The Netherlands; ^2^Section Electron Microscopy, Department of Cell and Chemical Biology, Leiden University Medical Center, The Netherlands

**Keywords:** alveolar type 2 cells, cell culture, emphysema, Organs-on-Chips, organoid

## Abstract

Development of effective treatment strategies for lung tissue destruction as seen in emphysema would greatly benefit from representative human in vitro models of the alveolar compartment. Studying how cellular cross talk and/or (altered) biomechanical cues affect alveolar epithelial function could provide new insight for tissue repair strategies. Preclinical models of the alveolus ideally combine human primary patient-derived lung cells with advanced cell culture applications such as breathing-related stretch, to reliably represent the alveolar microenvironment. To test the feasibility of such a model, we isolated primary alveolar type 2 cells (AEC2s) from patient-derived lung tissues including those from patients with severe emphysema, using magnetic bead-based selection of cells expressing the AEC2 marker HTII-280. We obtained pure alveolar feeder-free organoid cultures using a minimally modified commercial medium. This was confirmed by known AEC2 markers as well as by detection of lamellar bodies using electron microscopy. Following (organoid-based) expansion, cells were seeded on both cell culture inserts and the Chip-S1 Organ-Chip that has a flexible polydimethylsiloxane (PDMS) membrane enabling the application of dynamic stretch. AEC2s cultured for 7 days on inserts or the chip maintained expression of HTII-280, prosurfactant protein C (SP-C), SP-A and SP-B, and zonula occludens-1 (ZO-1) also in the presence of stretch. AEC2s cultured on the chip showed lower expression levels of epithelial-mesenchymal transition-related vimentin expression compared with static cultures on inserts. The combination of a straightforward culture method of patient-derived AEC2s and their application in microfluidic chip cultures supports successful development of more representative human preclinical models of the (diseased) alveolar compartment.

## INTRODUCTION

Chronic obstructive pulmonary disease (COPD) is one of the leading causes of death worldwide (1). Due to a lack of available curative treatments, combined with persistent and long-term effects of smoking, the alarming levels of air pollution, as well as aging of the population, this is not expected to improve in the near future (2). Chronic airway inflammation and progressive destruction of the alveolar compartment of the lungs (emphysema) are principal hallmarks of this disease (3, 4). Alveolar tissue destruction reduces the gas exchange capacity and elastic recoil of the lungs, thereby progressively affecting quality of life of these patients. Smoking is one of the main risk factors for development of COPD. In addition to smoking cessation and pulmonary rehabilitation, pharmacological treatment for COPD is largely limited to the use of long-acting β2-agonists (LABA), long-acting muscarinic antagonists (LAMA), and inhaled corticosteroids, which do not modify the course of the disease (5, 6). Lung volume reduction surgery (LVRS) can give alleviation for patients as it improves breathing by resection of the most damaged part of the lungs. Lung transplantation is the remaining treatment strategy when the decrease in lung function capacity becomes life threatening, but this is limited by the availability of matching donor lungs and rejection of transplanted lungs.

Development of new treatments that could halt or reverse the destruction of the alveolar compartment or potentially induce regeneration of lung tissue is reliant on human preclinical models of the alveolar compartment, and available alternatives such as (tumor-derived) cell lines or animal models, unfortunately, provide limited translation to in vivo human biology (7). Human induced pluripotent stem cells (hiPSC) offer an alternative source; however, generation of alveolar type 2 cells (AEC2s) from hiPSC is currently still time consuming, costly, and the hiPSC-derived alveolar cells generally display an immature phenotype (8).

Isolation protocols of human AEC2s were until recently complex procedures that were generally time consuming, required specialized equipment and/or complex cell culture media (9, 10), and carried a risk of contamination with mesenchymal cells. Furthermore, when AEC2s are propagated in conventional in vitro cell culture, they tend to differentiate over time to alveolar epithelial type 1 cells (AEC1s) (11). Finally, since culture-based expansion of AEC2s and, therefore, the yield of AEC2s is limited, the number of isolated cells restricts the experimental set-up. Recently, new methods have been published that overcome many of these limitations, demonstrating the isolation of murine or human AEC2s from lung tissue and subsequent expansion in organoid culture (12–14). These important improvements have already shown to be a great step forward, and a logical next step is to apply primary AEC2s, preferably patient-derived, in preclinical models of the alveolar compartment that include additional aspects of the alveolar environment, such as capillary blood flow and strain caused by breathing motions, as these mechanical forces are highly relevant in the alveolus environment but currently understudied (15). The development of Organs-on-Chips that recapitulate the tissue architecture more closely and allow integration of the dynamic biomechanical changes and cellular interactions within tissues further accelerates the development of relevant preclinical human in vitro models (16, 17).

Here we used a straightforward two-step protocol for AEC2 isolation from resected human lung tissue derived from nonsmokers, ex-smokers, smokers and emphysematous tissue from LVRS. Dissociation of the lung tissue was followed by a HTII-280-based enrichment step of AEC2s using magnetic beads. This isolation procedure was combined with a feeder-free organoid-based culture method using a minimally modified commercial medium that allows expansion and propagation of AEC2s for weeks up to several months, while maintaining AEC2 characteristics and preventing contamination by other cells. Once sufficiently expanded, cells were successfully used for culture on conventional cell culture inserts and innovative Organs-on-Chips, in which we demonstrated feasibility of using these patient-derived organoid-expanded AEC2s to develop models of the (diseased) alveolus in which the effect of cyclic stretch on AEC2 phenotype can be studied.

## METHODS

### Isolation, Maintenance, and Expansion of Primary Alveolar Type 2 Cells

#### Tissue processing.

AEC2s were isolated from tumor-free tissue from patients undergoing lung resection surgery for lung cancer (smokers, ex-smokers, and nonsmokers) or from emphysematous tissue from patients undergoing lung volume reduction surgery (LVRS) at the Leiden University Medical Center (LUMC, The Netherlands) for emphysema. Patient characteristics are summarized in Table 1. The use of surplus lung tissue for research following surgery was within the framework of patient care and in line with the “Human Tissue and Medical Research: Code of conduct for responsible use” (2011; www.federa.org) and followed advice of the LUMC Medical Ethical Committee. Tissue donation was based on a no-objection system for coded anonymous use of waste tissue, left-over from diagnostic or therapeutic procedures. “No-objection” negates the need for individual informed consent. All methods were carried out in accordance with relevant guidelines and regulations. The lung tissue homogenate preparation procedure was adapted from Witherden et al. (9). Resected lung tissue was cut into pieces of ∼5 cm^3^ and injected with 7.5 mL trypsin (1:250; 0.25% wt/vol; Gibco) in Hanks’ Balanced Salt Solution (HBSS; Thermo Fisher, Waltham, MA) and incubated for 15 min at 37°C; this was repeated for a total incubation time of 45 min. Trypsin activity was inhibited by injecting the tissue with 7.5 mL soybean trypsin inhibitor (SBTI; 0.1% wt/vol in HBSS; Sigma-Aldrich, St. Louis, MO). Next, tissue was manually cut as small as possible during max. 10 min at room temperature (RT). The processed tissue was collected in gentleMACS C tubes (Miltenyi, Leiden, The Netherlands) and ran twice on the gentleMACS tissue dissociator program M_lung_02.01 (Miltenyi) for further processing. This homogenate was then passed through a metal sieve to remove the biggest remaining pieces of tissue and next through a strainer with a mesh size of 100 µm (VWR International, Amsterdam, the Netherlands) to obtain a single cell suspension. The cells were centrifuged for 5 min at 265 *g* and supernatant was removed. When necessary (sample dependent), a red blood cell lysis (Miltenyi) was performed. To this end, the cell pellet was resuspended in 1 mL magnetic-activated cell sorting (MACS) buffer consisting of PBS, BSA (0.1% wt/vol; Sigma-Aldrich), and 2 mM EDTA (Thermo Fisher) to continue with HTII-280^+^ selection.

**Table 1.** Patient characteristics of resected lung tissue from lobectomy surgery or lung volume reduction surgery

	Lobectomy	LVRS
Number of donors	19	16
Male/female	12/7	4/12
Age (yr) mean [SD]	63.1 [11.2]	59.2 [4.7]
BMI mean [SD]	27.0 [6.2]	25.3 [3.6]
Smoking status (nonsmokers/ex- smokers/smokers)	3/6/7^#^	0/16/0
Pack·years (ex-smokers/smokers) [SD]	18.1 [15.3]/42.40 [9.56]^##^	28.3 [6.0]/-
FEV1 % pred [SD]	91.7 [19.7]^###^	31.5 [8.5]
DLCO % pred [SD]	79.4 [24.8]^#^	45.1 [12.3]

^#^ Data available from 16 lobectomy patients. ##Smokers data available from 12 current or ex-smoking lobectomy patients. ###Data available from 17 lobectomy patients. BMI, body mass index; DLCO, diffusion capacity of the lungs for carbon monoxide; FEV1, forced expired volume in 1 s; LVRS, lung volume reduction surgery.

#### HTII-280^+^ selection.

HTII-280^+^ AEC2s were isolated using a HTII-280 monoclonal mouse IgM antibody (Terrace Biotech, San Francisco, CA). Total tissue homogenate in 1 mL of MACS buffer was incubated with 25 µL of undiluted HTII-280 antibody for 15 min at 4°C. Following 5 min centrifugation at 265 *g*, cells were incubated with magnetic bead-labeled anti-mouse IgM (Miltenyi) for 15 min at 4°C, and subsequently, MACS selection was performed according to manufacturer’s instruction (Miltenyi).

#### Culture of alveolar type 2 cells in organoids.

HTII-280^+^ cells were collected by centrifugation at 265 *g* for 5 min. AEC2s were counted in trypan blue and resuspended in cold Basement Membrane Extract (BME2, Cultrex, Gaithersburg, MD). AEC2s (1 × 10^5^ viable cells/30 µL droplet/well) were seeded in a 48-well plate, after which droplets were allowed to solidify at 37°C. After 10 min, 500 µL complete alveolar organoid medium was added to the well. Alveolar cell culture medium consists of alveolar medium (Sciencell, Carlsbad, CA) with all supplements from the media kit except the antibiotics, which were replaced by Primocin (Invivogen, San Diego, CA). Alveolar cell culture medium supplemented with 4 µM CHIR99021 (CHIR; Sigma-Aldrich) is further referred to as “complete alveolar organoid medium.” At the start of culture or directly on passaging (until next medium refreshment), the complete alveolar organoid medium was supplemented with 10 µM Y-27632 (Cayman Chemical, Ann Arbor, MI). Medium was refreshed twice a week, and cells were passaged approximately every 2 wk adapting the passaging time to the donor proliferation rate. For passaging, medium was aspirated, and cold DPBS + EDTA (2.5 mM) was added to the well to dissolve the BME2. The organoid suspension was collected and centrifuged. Then the pellet was dissociated for max 5 min with TripLE Select Enzyme (Thermo Fisher) at 37°C to further dissolve the BME and dissociate the organoids. After that, SBTI was added to stop the trypsin activity and organoids were further dissociated into fragments by resuspension. The disrupted organoids were centrifuged, resuspended in BME2, and replated. Organoids were subsequently grown under standard cell culture conditions (37°C and 5% CO_2_).

### Cytospin Preparations

To validate the success of HTII-280^+^ isolation using magnetic beads, cytospin preparations were obtained from the lung tissue homogenate single-cell suspension before selection (unsorted) and after HTII-280^+^ selection, also including the negative fraction (flow through). Cytospin preparations were fixed using 4% formaldehyde (Sigma-Aldrich) in PBS for subsequent immunofluorescence staining.

### Electron Microscopy

To collect the organoids, medium was aspirated and cold DPBS + EDTA (2.5 mM) was added to the well to dissolve the BME2. The organoid suspension was centrifuged and the organoids were fixed for 60 min at RT with glutaraldehyde (1.5% vol/vol) in 0.1 M cacodylate buffer (pH 7.4) by adding an equal volume of double-concentrated fixative. After washing with 0.1 M cacodylate buffer, the organoids were treated with osmium tetroxide (1% wt/vol) and potassium ferrocyanide (1.5% wt/vol) in 0.1 M cacodylate buffer for 60 min at 4°C, and then washed with milliQ water. The organoids were then stained with 0.5% ruthenium tetroxide for 30 min at 4°C. After washing with milliQ water and pelleting in agar (3% wt/vol) in milliQ water, small blocks containing the organoids were excised, dehydrated in a graded ethanol series, and embedded in epoxy resin (LX-112; Ladd Research, Williston, VT). Ultrathin sections (100 nm thick) were cut and placed on mesh-50 or 100 copper EM grids covered with a carbon-coated Pioloform layer and poststained with 7% uranyl acetate and Reynolds lead citrate. The samples were examined in a BioTwin Tecnai 12 electron microscope (Thermo Fisher) operated at 120 kV and equipped with a 4 K Eagle CCD Camera (Thermo Fisher).

### Cell Cultures on Inserts

Cell culture inserts (Corning; membrane pore-size 0.4 µm, PET membrane) were coated with a mixture of 30 μg/mL bovine collagen I (PureCol; Advanced BioMatrix, San Diego, CA), 10 μg/mL BSA (Thermo Fisher), and 10 μg/mL fibronectin (Promocell, PromoKine, Bio-Connect, Huissen, The Netherlands) in PBS and seeded with either the HTII-280^+^ lung cells directly isolated from tissue (P0) or with the dissociated AEC2s from the organoids at various passages. Alveolar medium was added to the basal and apical compartment of the inserts and refreshed twice a week. After 7 days in culture, the inserts were fixed for immunofluorescence staining using 4% formaldehyde in PBS.

### Alveolus Lung-Chip Cultures

Both channels of the commercial Chip-S1 (Emulate Inc, Boston, MA) were activated using the provided reagents ER-1 and ER-2. In short, ER-1 was resuspended in 5 mL ER-2 buffer and directly pipetted into both channels of the chip. Next, the chips were placed under UV light for 10 min followed by two washes of both channels with ER-2, followed by another round of ER-1, UV, and ER-2 washes. After the second ER-2 wash, the channels were washed with PBS. Next, both channels were filled with 300 µg/mL human collagen IV (Sigma-Aldrich) solution in PBS and incubated overnight at 37°C and 5% CO_2_ to allow deposition of the collagen on the membrane in the chip [membrane pore-size 7 µm, polydimethylsiloxane (PDMS) membrane].

Next, both channels were washed with complete alveolar organoid medium before cells were seeded in the top channel. To seed sufficient AEC2s, 3 × 90 µL organoid-containing drops of AEC2s were collected per chip, and organoids were dissociated as described when passaging the organoids. The cell pellet was resuspended in complete alveolar organoid medium and 30 µL of this cell suspension was used to seed the top channel of one chip. Cells were left to adhere for ∼6 h in the incubator in presence of 10 µM Y-27632. Next, top and bottom channels were infused with prewarmed complete alveolar organoid medium containing 10 µM Y-27632, and chips were connected to prewarmed media-filled fluidic manifolds, named “Pods” (Emulate Inc.). After obtaining liquid-liquid interface connection between chips and pods, these units were placed in the microperfusion instrument, named “Zoë” (Emulate Inc.). After finishing the initial regulate cycle program, the chips were continuously perfused at a flow rate of 30 µL/h in both the top and bottom channel. Approximately 24 h after this first regulate cycle program, a so-called via-wash was performed, dislodging any bubbles in the pods reservoir channel openings, followed by a second regulate cycle. Alveolar medium in the pods was refreshed after 48 h (no addition of Y-27632), which was repeated every other day throughout the time of culture (all without Y-27632). When the cultures reached confluence (usually between ∼2 and 4 days postseeding), chips were divided into two groups: control (flow rate 30 µL/h top and bottom channel flow conditions) and stretch (30 µL/h top and bottom channel flow conditions + 10% stretch at 0.25 Hz) (18).

### Imaging of Cytospin Preparations, Organoids, Cell Culture Inserts, and Chips

#### Cytospin preparations and cell culture inserts.

Fluorescent staining was performed on cytospin preparations, cells cultured on inserts, and paraffin sections of the organoids according to the following protocol. After fixation, cells were incubated with permeabilization and blocking buffer [1% wt/vol BSA, 0.3% vol/vol Triton X-100 (Sigma-Aldrich) in PBS] for 30 min at 4°C. Paraffin sections were pretreated with DAKO pH 9 antigen retrieval solution according to manufacturer’s instruction (DakoCytomation, Denmark, Glostrup). The primary antibody (Table 2) was added in blocking solution to the cells for 1 h at RT. Next the samples were washed with PBS and incubated with fluorescent-labeled secondary antibody together with 4′,6-diamidino-2-phenylindole (DAPI; Sigma-Aldrich) for 30 min at RT.

**Table 2.** Antibodies

Primary Antibody	Species	Company	Cat. No.	Dilution
HTI-56	Mouse	Terrace Biotech	TB-29AHT1-56	1:50
HTII-280	Mouse	Terrace Biotech	TB-27AHT-280	1:200
Surfactant protein A	Rabbit	Merck Millipore	AB3420-I	1:100
Surfactant protein B	Mouse	Invitrogen	MA1-204	1:100
Prosurfactant protein C	Rabbit	EMD Millipore	Ab3786	1:100
E-cadherin	Mouse	BD Transduction Laboratories	610182	1:400
RAGE 8G4	Rabbit	Bioss	50-198-9703	1:400
Aquaporin 5	Rabbit	EMD Millipore	178615	1:400
Keratin 5	Rabbit	Abcam	ab52635	1:200
Zonula occludens-1	Mouse	Thermo Fisher	339100	1:100
Vimentin (Clone V9)	Mouse	Agilent Dako	M0725	1:100

Secondary Antibody	Species	Company	Cat. No.	Dilution
Alexa Fluor 448 donkey anti-mouse	Mouse	Thermo Fisher	A21202	1:200
Alexa Fluor 568 donkey anti-mouse	Mouse	Thermo Fisher	A10037	1:200
Alexa Fluor 448 goat anti-rabbit	Rabbit	Thermo Fisher	A11008	1:200
Alexa Fluor 568 donkey anti-rabbit	Rabbit	Thermo Fisher	A10047	1:200

#### Organoids.

After dissolving the droplets with recovery solution (Corning, Corning, NY), the alveolar organoids were fixed in 4% formaldehyde in PBS for 30 min. Next the organoids were taken up in low-melting agarose (2% wt/vol; ThermoFisher) and embedded in paraffin. Slices of 4 µm were cut and the organoids were stained with various antibodies (Table 2) for either immunostainings, using fluorescence or the chromogen NovaRED (Vector Laboratories), or hematoxylin-eosin (H&E) staining. For stainings performed on paraffin sections, samples were pretreated for antigen retrieval using DAKO pH 9 antigen retrieval solution. One slice was assessed per staining, containing generally between 20 and 30 organoids.

#### Lung-Chips.

Alveolar cells in the chips were washed with PBS and fixed using 4% formaldehyde, which was pipetted into both channels and incubated for 20 min at RT, washed with PBS, and stored in PBS at 4°C until staining. Chips were subsequently removed from the carrier and cells were blocked and permeabilized using 0.5% (vol/vol) Triton X-100 in PBS with 5% (wt/vol) BSA for 1 h at RT. Chips were cut into two pieces using a razor blade, and channels were filled with primary antibodies (Table 2) diluted in the blocking buffer and incubated for 1 h at RT. Samples were rinsed three times for 5 min with PBS. Next, channels were filled with secondary antibodies diluted in blocking buffer that was left incubating for 1 h at RT, followed by a 3 × 5 min wash with PBS. DAPI was used to stain cell nuclei. Channels were next filled with ProLong gold antifade (Thermo Scientific) and stored in the dark at 4°C until imaging. Chips were imaged on coverslips 25 × 75 mm (Bellco Glass, Vineland, NJ, via Electron Microscopy Sciences, Hatfield, PA) with 0.13–0.17 mm thickness using a Leica DMi8 microscope (Leica microsystems, Wetzlar, Germany) equipped with an Andor Dragonfly 200 spinning disk confocal (Andor Technology, Belfast, UK) using a ×10 objective (NA 0.30), ×20 water objective (NA 0.50) or ×40 water objective (NA 0.80).

#### Vimentin quantification.

ImageJ software (NIH, Bethesda, MD) was used to analyze the fluorescence images with vimentin staining. Per donor 5 separate images with per image a 500 µm^2^ field were analyzed and the number of nuclei and vimentin positive cells were counted.

### RNA Isolation, cDNA, and qPCR Analysis

Alveolar cells in the chip and inserts were lysed by the addition of 200 µL lysis buffer from the RNA tissue isolation kit (Promega Benelux B.V., Leiden, The Netherlands) per insert. For the chip, a p200 filter tip was inserted on one side of the top channel. Next cells were lysed by the addition of 100 µL of lysis buffer in the top channel and the pipette tip was left in the other side of the channel. After ∼2 min, the lysis buffer was collected and another 100 µL was added to the same chip and pooled with the previous 100 µL of lysate. Lysates were stored at −20°C until RNA isolation. RNA isolation and conversion to cDNA were performed as previously described (19). RNA (100 ng) was converted to cDNA. Quantitative PCR (qPCR) reactions were performed in triplicate using a CFX-384 Real-Time PCR detection system (Bio-Rad Laboratories, Veenendaal, The Netherlands), IQ SYBRGreen supermix (Bio-Rad), and primes (Table 3). Expression was normalized for the geometric mean of the reference genes *OAZ1* and *ATP5B.*

**Table 3.** Primers

Gene	Encoding Protein	Sequence Forward Primer	Sequence Reverse Primer
*OAZ1*	Ornithine decarboxylase antizyme 1	GGATCCTCAATAGCCACTGC	TACAGCAGTGGAGGGAGACC
*ATP5B*	ATP synthase F1 subunit β	TCACCCAGGCTGGTTCAGA	AGTGGCCAGGGTAGGCTGAT
*SFTPB*	Surfactant protein B	CTTCCAGAACCAGACTGACTCA	GCTCGGAGAGATCCTGTGTG
*SFTPC*	Surfactant protein C	CTCTCTGCAGGCCAAGCCCG	TTCCACTGACCCGGAGGCGT
*AQP5*	Aquaporin 5	ATCTGGCCGTCAACGCGCTC	AGTGAGCGGGGCTGAACCGA
*PDPN*	Podoplanin	AACCAGCGAAGACCGCTATAA	CGAATGCCTGTTACACTGTTGA
*VIM*	Vimentin	TTGAACGCAAAGTGGAATC	AGGTCAGGCTTGGAAACA
*FN1*	Fibronectin	TGGAGGAAGCCGAGGTTT	CAGCGGTTTGCGATGGTA

### Statistics

Statistical analysis was performed using Graphpad Prism version 9 (GraphPad Software Inc. La Jolla, CA). Data are depicted as individual data points with SE and significance was tested using a paired *t* test, and differences were considered significant at *P* < 0.05.

## RESULTS

### Generation of Alveolar Type 2 Organoid Cultures from Homogenized Lung Tissue

**Figure 1.**
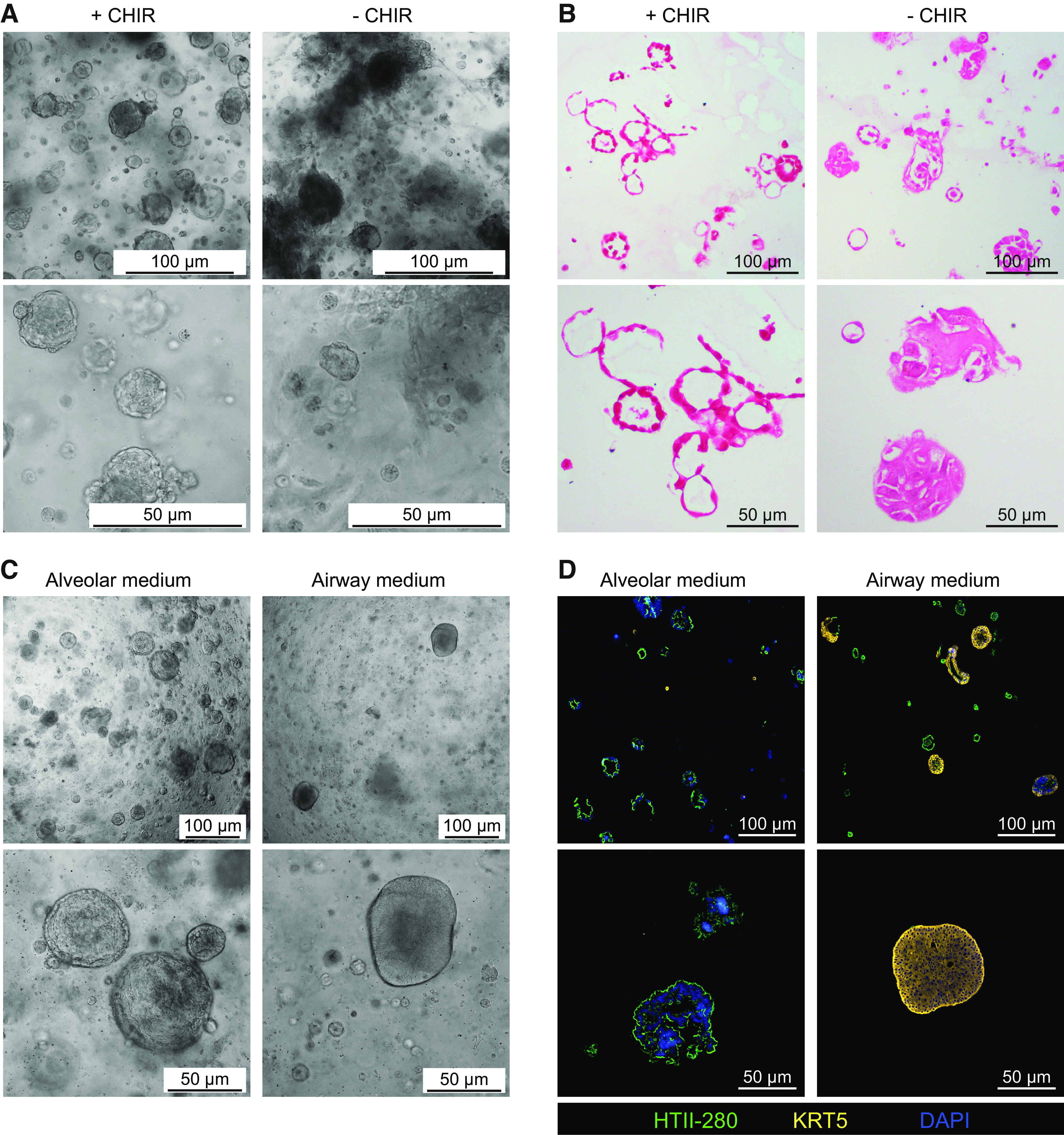
Generation of alveolar type 2 organoid cultures from homogenized lung tissue. Lung tissue was enzymatically digested to obtain a single-cell suspension which was subsequently cultured in BME2 drops. *A*: bright-field imaging of organoids cultured in alveolar medium supplemented with (+) or without (−) 4 µM CHIR99021 (CHIR; a GSK3 inhibitor). *B*: bright-field images of H&E staining of organoids cultured in alveolar medium supplemented with (+) or without (−) CHIR. Representative images of experiments performed with *n* = 3 different donors (*A* and *B*). *C*: bright-field images of organoids cultured from lung tissue homogenate in alveolar medium (including CHIR) or airway organoid medium. *D*: fluorescent imaging of organoids stained for HTII-280 (AEC2s, green) and keratin 5 (KRT5; airway basal cells, yellow), nuclei were stained with DAPI (blue). Representative images of experiments performed with *n* = 3 different donors. One paraffin slice was assessed per donor per staining, containing generally between 20 and 30 organoids. AEC2s, alveolar type 2 cells; H&E, hematoxylin-eosin.

We developed a straightforward procedure for isolation of AEC2s from human lung tissue, with a similar approach as recently reported by another group using healthy tissue (14). We dissociated macroscopically normal lung tissue derived from lung cancer patients with or without a smoking history (current smokers, ex-smokers, and nonsmokers) and additionally, to our knowledge for the first time, used emphysematous lung tissue from LVRS surgery and obtained a single cell suspension from these tissues using protease digestion. We subsequently cultured this single-cell suspension in BME2 droplets to develop organoids. AEC2 organoid formation was rarely observed unless the commercial alveolar medium was combined with the canonical WNT activator CHIR99021 (CHIR; a GSK3 inhibitor). This combination was pivotal for formation of predominantly alveolar organoids in P0 and pure alveolar cultures after the first passage (Fig. 1, *A* and *B*). To confirm that the use of this specialized medium was sufficient to facilitate AEC2 organoid formation from the total lung homogenate suspension, we next cultured the total lung homogenate suspension either in our complete alveolar organoid medium or in specialized airway organoid medium (20). When cells from the homogenate were cultured in complete alveolar organoid medium, the organoids generated were characterized by presence of the alveolar marker HTII-280 and absence of the airway basal cell marker keratin 5 (HTII-280^+^/KRT5^−^), whereas using the airway organoid medium, the development of predominantly airway organoids was observed (HTII-280^−^/KRT5^+^; Fig. 1, *C* and *D*). HTII-280^+^-cell clumps were visible in these airway organoid medium cultures; however, they remained small and did not form organoids. Despite developing a medium that successfully supports alveolar organoid formation, for some donors, organoid formation was limited or unsuccessful. Characterization of the HTII-280^+^ population in the lung homogenate suspension using cytospin preparations, showed a strong donor-dependent ratio of AEC2s to other (HTII-280^−^) cells. We therefore hypothesized that as a result, the AEC2s to other cell ratio per gel drop was insufficient in some donors for successful establishment of alveolar organoids. To overcome this issue, an enrichment step for AEC2s was included.

### Alveolar Type 2 Cell Enrichment from Peripheral Lung Tissue Homogenate by HTII-280^+^ Selection

**Figure 2.**
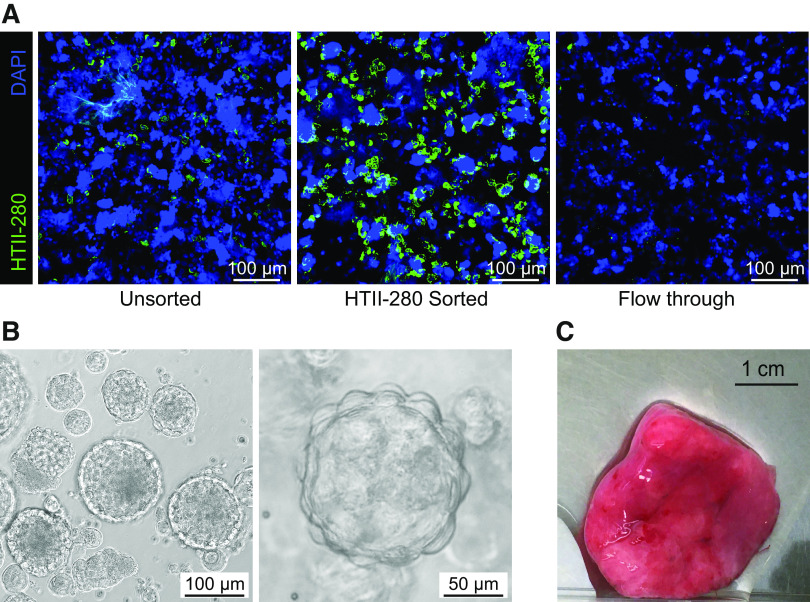
HTII-280^+^ selection of alveolar type 2 cells from homogenized lung tissue. Lung tissue was enzymatically digested to obtain a single-cell suspension, after which HTII-280 selection via magnetic beads was performed. Sorted cells were subsequently cultured in BME2 drops. *A*: representative immunofluorescent images of cytospin preparation of lung tissue homogenate (unsorted, sorted, and the flow-through) after sorting. AEC2s were stained with HTII-280 (green) and DAPI (blue). Representative image from *n* = 3 independent experiments with different donors. *B*: representative bright-field images of organoids derived from the HTII-280^+^ fraction cultured in complete alveolar medium. Representative image from *n* = 4 independent experiments with different donors. *C*: example of amount of lung tissue that is minimally required to isolate sufficient cells for AEC2 organoid development. AEC2s, alveolar type 2 cells; BME2, Basement Membrane Extract.

Our initial studies indicated that cell cultures started from the whole lung homogenate caused issues with obtaining sufficient AEC2s for organoid formation in some donors. Therefore, an enrichment step was included for AEC2s. Isolation of AEC2s from peripheral tissue either by selection via adherence steps or EpCAM^+^ epithelial cell sorting results in an undesired mixture of cell types, including AEC2s but also airway epithelial cells and mesenchymal cells. A decade ago, Gonzalez et al. (21) showed that the AEC2 marker HTII-280 could be used to increase the purity of the AEC2 population. This method was based on fluorescence-activated cell sorting (FACS), which requires expensive equipment, skilled personnel, and is time-consuming. Here we decided to integrate a magnetic bead-based isolation that has been successfully used to isolate AEC2s (14). Lung tissue homogenate was incubated with a HTII-280 antibody and next coupled to anti-mouse IgM-coated magnetic beads to allow magnetic bead-based AEC2 isolation. Although, depending on the donor, not always 100% pure AEC2 populations could be obtained using this method, the enrichment was clear for all donors (example in Fig. 2*A*). Using this method, in combination with our complete alveolar organoid medium, we could consistently establish AEC2 organoid cultures (Fig. 2*B*), while needing only relatively small pieces of tissue (Fig. 2*C*). We obtained a ∼85% success rate using the finalized method for the formation of alveolar organoids up to P1. This method of isolation proved successful when using peripheral tissue obtained from macroscopically normal lung tissue from lung cancer surgery with or without a smoking history or COPD. In addition, AEC2 isolation from emphysematous lung tissue removed during LVRS for severe emphysema was successful in ∼70% of isolations, using different versions of the protocol but successfully validated with the final protocol presented in the methods section.

### Propagation and Expansion of Primary Alveolar Type 2 Cells in Feeder-Free Organoid Cultures

**Figure 3.**
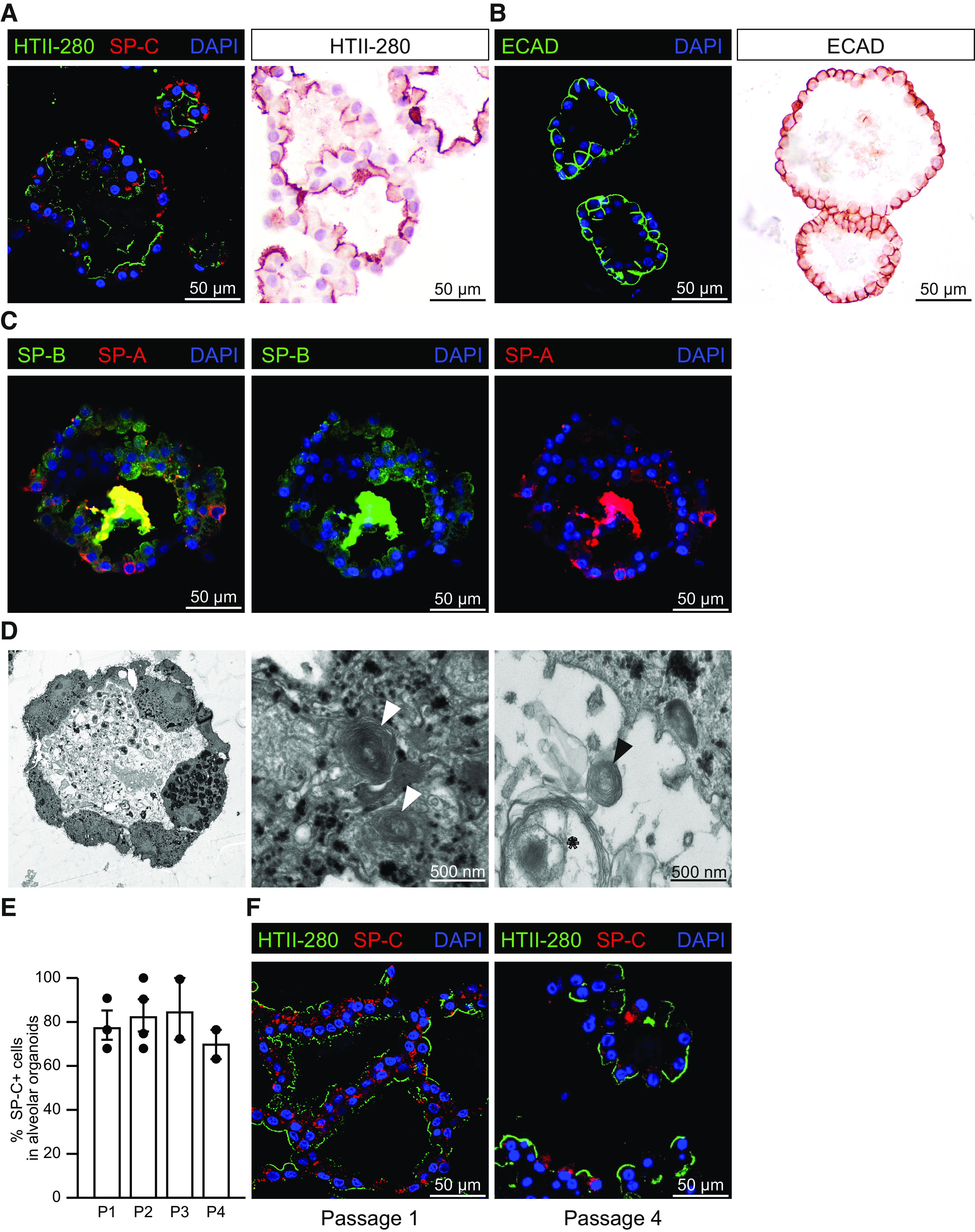
Propagation and expansion of primary alveolar type 2 cells in vitro. *A*: fluorescent image of organoids stained for HTII-280 (AEC2s, green) and prosurfactant protein C (SP-C, red) and nuclei were stained with DAPI (blue); immunohistochemical (IHC) staining for HTII-280. Representative images of *n* = 4 different donors. *B*: organoid staining for E-cadherin (ECAD, green); fluorescence (*left*) and IHC (*right*). *C*: representative immunofluorescent images of a cross section of embedded AEC2 organoids stained with surfactant protein A and B (SP-A, red; SP-B, green) in overlay (*left*), or separated for SP-B (*middle*) or SP-A (*right*). *D*: electron microscopic image of embedded AEC2 organoid. Arrows are used to indicate intracellular lamellar bodies (white arrows), extracellular lamellar bodies (black arrows) as well as an uncoiled extracellular lamellar body (asterisk). *E*: quantification of SP-C^+^ cells in organoids cultured at different passages (P#). *F*: example immunofluorescent image of a cross-section of embedded AEC2 organoids after *passage 1* and after *passage 4* showing loss of SP-C. Cells are stained for HTII-280 (AEC2s, green) and surfactant protein C (SP-C, red) and nuclei with DAPI (blue). *n* = 2–4 different donors. One paraffin slice was assessed per donor per staining, containing generally between 20 and 30 organoids. AEC2s, alveolar type 2 cells.

After AEC2 enrichment by HTII-280^+^ selection, the cells were cultured in BME2 to allow organoid formation with the aim of propagating the AEC2s to obtain sufficient cells for experiments. We cultured the isolated AEC2s in a feeder-free organoid system and could propagate the AEC2 organoids for weeks to months, depending on the donor, before cessation of organoid growth. It remains unclear which factor(s) play(s) a role in cessation of organoid growth. On average ∼80% of cultures that reached P1 were also able to reach passage P3. During passaging, AEC2s in the organoids maintained their AEC2 characteristics, including expression of HTII-280, SP-C (Fig. 3*A*), and E-cadherin (Fig. 3*B*) and remained negative for AEC1 markers RAGE and Aquaporin 5 or epithelial-to-mesenchymal transition (EMT) marker vimentin (Supplemental Fig. S1, all Supplemental material is available at https://doi.org/10.6084/m9.figshare.17125061). Additionally, mature surfactant protein A and B (SP-A and SP-B) were readily expressed intracellularly and secreted in the organoid lumen (Fig. 3*C*). Since the SP-C antibody is directed to prosurfactant protein C, we could not assess secretion of mature/processed SP-C in the lumen of the organoid. However, we confirmed the presence of lamellar bodies in the AEC2s in organoid culture by electron microscopy (Fig. 3*D*). Interestingly, also intact lamellar body-like structures could be detected in the organoid lumen (Fig. 3*D*, *right*).

In general, AEC2s were used for experiments between P2 and P5. HTII-280 expression remained positive for all cells in the organoids over time, and also cultures from most donors maintained SP-C expression (Fig. 3*E*), although SP-C expression decreased in organoids from some donors (example shown in Fig. 3*F*).

Unexpectedly, propagation and expansion rates were similar between macroscopically normal lung tissue and LVRS tissue-derived AEC2s (observation not quantified), despite the fact that LVRS-resected tissue constitutes the most damaged part of the emphysematous lung.

### Culturing Alveolar Type 2 Cells on Cell Culture Inserts and Chips

The unique design of the chip allows for the implementation of cyclic stretch on the alveolar cell cultures (22, 23). Especially when regarding the lungs, the application of breathing biomechanics is highly relevant and so far studies have not focused on the use of primary patient-derived cells. To establish proof-of-principle that the primary AEC2s obtained via our isolation protocol could be cultured under cyclic stretch in the Lung-Chip and whether this affected their AEC2 phenotype, we dissociated the alveolar organoids and seeded them on chips or conventional cell culture inserts for comparison. The coating differed between inserts and chips since collagen IV coating did not result in successful attachment of the cells to the insert’s PET membrane whereas collagen I/fibronectin/BSA did, whereas in the chip, the opposite was observed. AEC2s seeded on coated inserts or chips were cultured in submerged conditions for 7 days. During the last final days of this 7-day period, in part of the chips stretch was applied. The level of cyclic stretch used was similar as previously reported (22) and mimics what is expected in an alveolus (24, 25).

**Figure 4.**
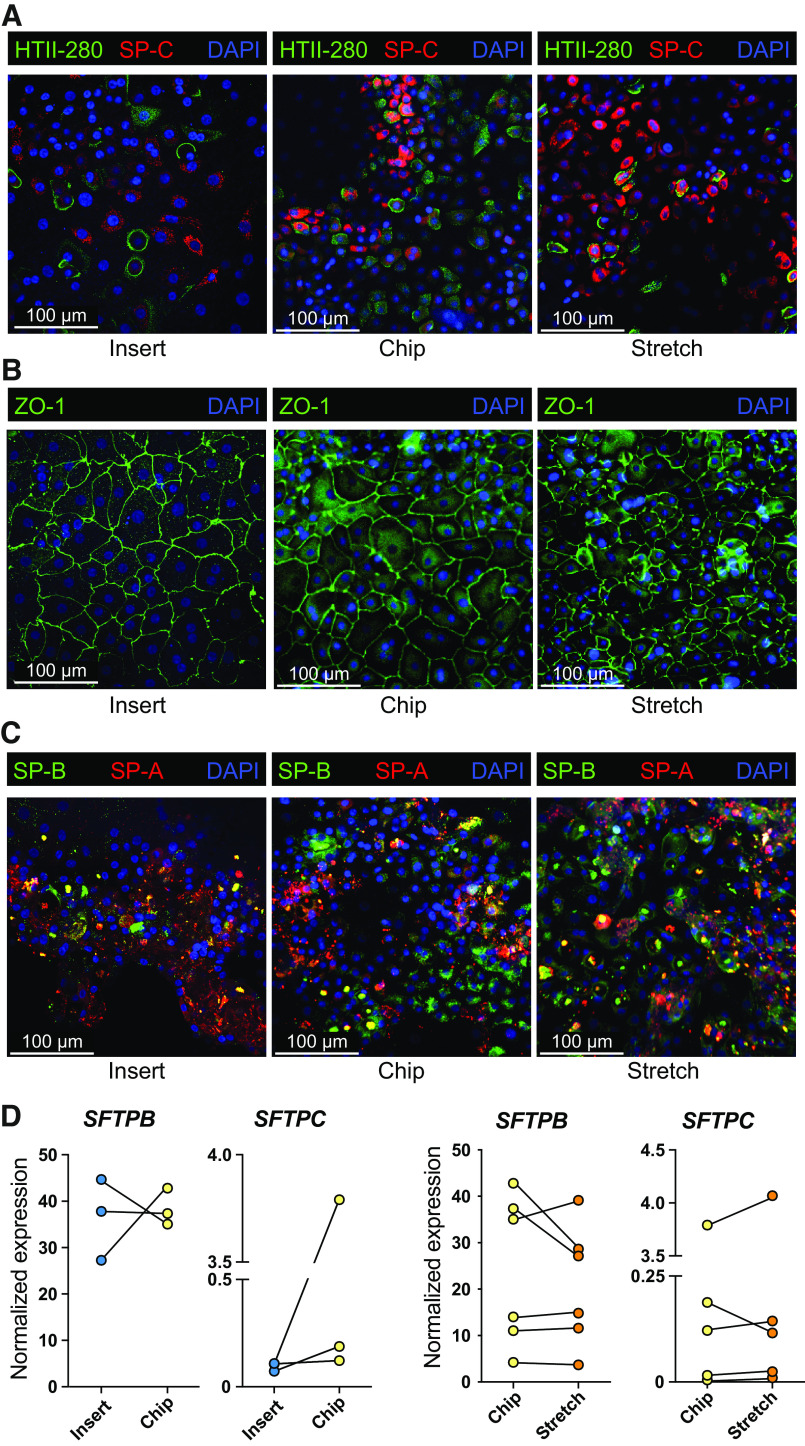
Alveolar type 2 cell culture after organoid-based expansion on insert and Alveolus Lung-Chip platform. Organoids from donors at different passages were dissociated, mixed and seeded on inserts or the Alveolus Lung-Chip and cultured for 7 days. *A*: fluorescent images of AEC2s after 7 days of culture on inserts or chips, in presence or absence of stretch. Cells were fixed and stained for HTII-280 (AEC2s, green), prosurfactant protein C (SP-C, red), and nuclei with DAPI (blue). Representative image from *n* = 4 independent experiments with different donor (mixes). *B*: the same cultures represented in *A* were furthermore stained with zonula occludens-1 (ZO-1, green), or *C*: surfactant proteins A (SP-A; red) or B (SP-B; green). *D*: at *day 7*, culture cells were lysed and RNA was isolated followed by cDNA synthesis to assess gene expression levels of surfactant protein B (*SFTPB*) and C (*SFTPC*). Data are shown as delta Ct normalized for the geometric mean of the reference genes *OAZ1* and *ATP5B*. Cultures on inserts are indicated with blue dots, chip control cultures with yellow dots and chip cultures exposed to 5 days of stretch with orange dots. The comparison between insert and chips was performed with three different donors. The comparison of stretch application with the control chip was performed with six different donor (mixes). AEC2s, alveolar type 2 cells.

Following culture, cells were analyzed for the presence of AEC2 and AEC1 phenotypic markers and tight junctions. We found that AEC2s cultured for 7 days on insert or on chip (the latter in the presence or absence of stretch) maintained expression of AEC2 markers HTII-280 and/or SP-C (Fig. 4*A*), and zonula occludens-1 (ZO-1) throughout the monolayer (Fig. 4*B*). Whereas HTII-280^+^ cells were present throughout the different cell cultures, we also observed a substantial number of cells that were negative for either AEC2 marker. When analyzing other surfactant proteins, that is, surfactant protein A and B (SP-A and SP-B), we observed that most cells in culture expressed SP-A and -B (Fig. 4*C*), whereas AEC1 markers HTI-56, Podoplanin, Aquaporin 5, or RAGE could not be reliably be detected on either protein (RAGE, HTI-56, Podoplanin) or gene level (*PDPN, AQP5*; data not shown), suggesting a more AEC2 phenotype rather than AEC1. We also quantified surfactant proteins B and C on mRNA level and found no significant difference in expression of these surfactants between insert or chip cultures or when stretch was applied (Fig. 4*D*).

### Assessment of Epithelial-to-Mesenchymal Transition in Insert and Chip Cultures

**Figure 5.**
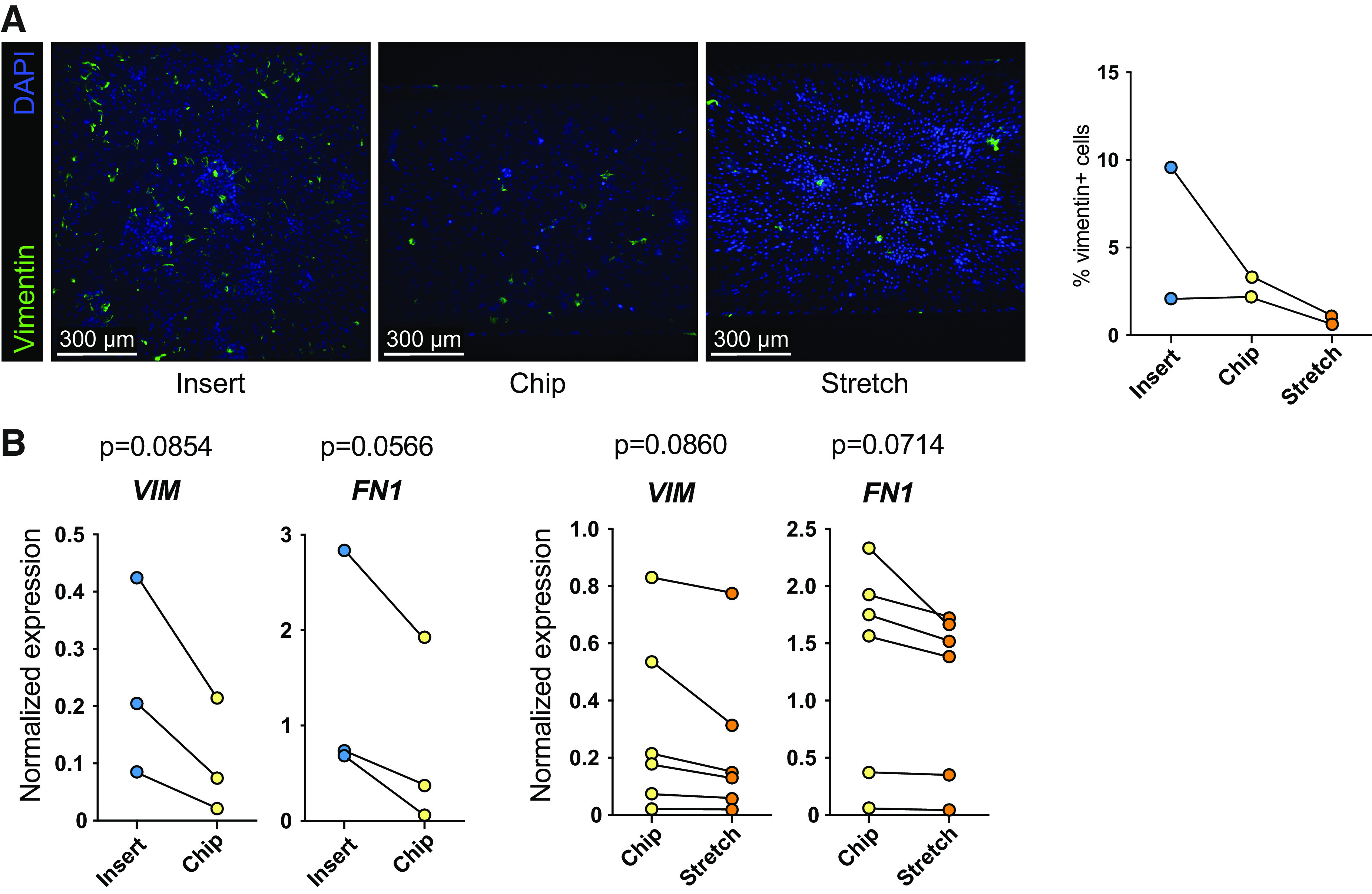
Epithelial-to-mesenchymal transition-related marker expression in alveolar type 2 cultures on inserts and chips. Organoids from donors at different passages were dissociated, mixed, and seeded on inserts or the Alveolus Lung-Chip for 7 days. *A*: fluorescent images of AEC2s after 7 days of culture on inserts or chips. Cells were fixed and stained for vimentin (AEC2s, green) and nuclei were stained with DAPI (blue). Image in which vimentin reduction was visible from *n* = 2 independent experiments with different donors. Quantification of vimentin staining (right graph). *B*: gene expression levels of vimentin (*VIM*) and fibronectin (*FN1*) in cell lysates of cultures on inserts, chips, or chips that were exposed to 10% stretch at 0.25 Hz for 5 consecutive days. Data are shown as delta Ct normalized for the geometric mean of the reference genes *OAZ1* and *ATP5B*. Cultures on inserts are indicated with blue dots, chip control cultures with yellow dots, and chip cultures exposed to 5 days of stretch with orange dots. The comparison between insert and chips was performed with *n* = 3 different donors. The comparison of stretch application with the control chip was performed with six donor (mixes). Significance was established using a paired *t* test; the level of significance was set at *P* < 0.05. AEC2s, alveolar type 2 cells.

To investigate whether epithelial-to-mesenchymal transition (EMT) contributed to the HTII-280^−^ cells in culture, we analyzed vimentin and fibronectin expression in cells cultured on inserts or chips. The level of vimentin expression was highly donor dependent, but overall relatively few cells were found to be vimentin positive (Fig. 5*A*); however, we did observe differences between cell cultures on insert or chip. Protein and gene expression level confirmed that cultures on chip showed a near significant reduction in vimentin and fibronectin (Fig. 5*B*), which was not further affected by stretch. Vimentin could not be detected in the alveolar organoids, suggesting the culture conditions in insert or chip promote vimentin expression. Despite that we did not observe notable effects of stretch on AEC2 phenotype, we did notice a slight change in morphology. Some cultures exposed to dynamic stretch displayed an elongated morphology perpendicular to the direction of the stretch, more pronounced in some donor (mixes) than others (Supplemental Fig. S2*A*).

## DISCUSSION

An essential component of a relevant model of the (diseased) alveolus environment is the inclusion of patient-derived AEC2s. Here we show that these cells can be isolated from relatively small pieces of lung tissue and, after organoid-based feeder-free expansion, can be cultured on traditional cell culture inserts and organ-chips. AEC2 isolation was successful both when using relatively healthy, unaffected lung tissue as well as from tissue from long-term smokers, patients with COPD, and highly emphysematous lung tissue that was obtained following LVRS.

When we cultured the cells from the complete whole lung tissue homogenate in airway organoid medium (20), we obtained predominantly KRT5^+^ airway organoids and many small alveolar cell clumps that did not develop further. Conversely, when the lung homogenate was cultured in complete alveolar organoid medium, analysis of the culture revealed little-to-no KRT5^+^ airway epithelial cells but predominantly HTII-280^+^ AEC2 organoids. These results highlight the possibility to culture (small) airway and alveolar epithelium from the same lung tissue homogenate and study spatial effects per donor in a controlled manner. Although these observations provide strong support for the selection pressure issued by these specialized media, we cannot exclude that any remaining non-AEC2s present after isolation and during the start of the culture may influence success of the alveolar cell cultures. We have tested further depletion of leukocytes via CD45 (data not shown), which did not affect AEC2 organoid formation.

The usefulness of HTII-280 as a marker for the magnetic bead-based enrichment of the AEC2 population was demonstrated previously (12, 14, 21) and greatly improved the success rate of organoid formation after isolation while maintaining a straightforward workflow for AEC2 isolation from human lung tissue. Although we obtained a pure alveolar organoid culture after the first passage of the organoids, we did find additional nonepithelial cells in the isolated population at the initiation of organoid culture (P0). However, these cells were no longer present in our culture after the first passage and there was no contamination with airway epithelial cells in the alveolar organoid cultures after the first passage.

Strikingly, the (severe) emphysematous tissue yielded comparable numbers of AEC2s, demonstrating the versatility of this protocol for use with both healthy and (very) diseased lung tissue. The lack of difference in yield, growth rate, or organoid forming capacity of the LVRS tissue-derived AEC2s compared with cells from other donors suggests that during emphysema development, possibly, a selection takes place of a more robust AEC2 subset from this tissue or alternatively the (favorable) culture conditions are compensating for any defects. Studies in mice show that chronic exposure to cigarette smoke increases the stemness of AEC2s (26), which may be a mechanism also operative in these tissues. It will be interesting to study if the capacity of AEC2s from these donors to differentiate toward AEC1 is affected and how application of stretch would influence this. Successful isolation from this diseased tissue now allows new experimental approaches to study repair and regeneration using cells derived from this unique microenvironment.

The established organoid cultures could be propagated for weeks up to months while maintaining expression of classical AEC2 markers (27) and secretion of surfactant proteins in the lumen of the organoids. Interestingly, when assessing the presence of lamellar bodies via EM technology, we observed intact lamellar body-like structures not only in the cells but also in the lumen of the organoid. Secretion or exocytosis of intact lamellar bodies and their subsequent conversion into tubular myelin and surfactant sheets is a unique and complex process (28). Secreted lamellar bodies are either converted into tubular myelin to form a surface active film/sheet or are recycled by re-uptake by the AEC2s (29). Our observation of luminal lamellar bodies is in line with literature data showing presence of lamellar bodies in the alveolar lumen in fetal rat lungs and can possibly be explained by the observation that air exposure, absent in the fetal lung lumen and the lumen of organoids, is a trigger for the transition of lamellar bodies to surfactant films (28, 30).

When we seeded these cells on inserts or chips, we observed that after 7 days of culture, HTII-280, SP-C, SP-A, and SP-B were detected throughout the culture, although in contrast to previous reports, we could not establish a transition to an AEC1 phenotype (31). It is unclear whether this is a technical issue with the staining procedure or whether AEC1 differentiation did not occur in our cultures. We can therefore not exclude the presence of AEC1s but we hypothesize that the addition of the WNT agonist CHIR to the complete alveolar culture medium contributes to the lack of differentiation towards AEC1s both in organoid cultures and in the chip (in presence or absence of stretch). The effect of canonical WNT activation on the propagation and AEC2 morphology has been described previously in organoid cultures (32). Furthermore, it was shown that medium containing human serum can direct differentiation from AEC2s to AEC1s in (organoid) culture (14), which should be addressed in future studies.

In our organoids, but also in the cultures on inserts and chip, we furthermore noticed that AEC2s contained HTII-280 and/or SP-C double- or single-positive cells, whereas most cells expressed surfactant SP-A and/or SP-B. The loss of SP-C is widely interpreted as a loss of AEC2 phenotype (11, 33); however, we demonstrate that these cells do not lose expression of the mature HTII-280 marker and/or other surfactant proteins. Transcriptomic analysis has revealed various subsets of alveolar cells, characterized by expression of subset-specific genes (34). In addition, Choi et al. showed that various alveolar cell subsets could be derived from AEC2s by exposure to (macrophage-derived) IL-1β in mice. It would be interesting to assess whether this approach also affects human AEC2 differentiation in culture and whether the AEC2s that do not stain positive for SP-C but do stain for the other AEC2 markers, represent subsets with specific functions or possibly transitional states.

Besides assessing AEC-related markers, we also investigated whether the cells in culture underwent EMT. We assessed vimentin protein expression and vimentin and fibronectin on gene expression and it was striking to see that AEC2 cultures on the chip had lower levels of vimentin compared with those on inserts. One possible explanation could be the higher initial seeding density used to seed the chips. Seeding density was slightly variable as these cells are obtained from disrupted organoids that do not form a completely single cell suspension at the time of seeding. We noticed that when AEC2s were seeded at a lower density, they quickly became larger in morphology (Supplemental Fig. S2*B*). Possibly, the difference in stiffness between the chips, which have a softer PDMS membrane, and the stiffer PET membrane in the inserts could account for this behavior. This would be in line with the increased awareness of the fact that culture substrate stiffness can influence cellular behavior (35). Cultures with lower seeding densities on chip that underwent dynamic stretch showed signs of cell detachment, suggesting that these forces on the cells may affect their adherence to the membrane.

We observed that the alveolar cells exposed to stretch slightly aligned in the direction of the flow and perpendicular to the direction of the stretch within 24 h after initiation of stretch. It is at present unclear whether this response mimics behavior of the alveolar cells in situ, especially as this response varied highly between donor (mixes). Extending the model to include additional cell types, thereby more closely mimicking the alveolar compartment, will be the logical next step to start answering questions related to the impact of biomechanical forces on lung alveolar biology.

A limitation of the method presented here, similar as those of others, is that the rate of proliferation of the AEC2s is relatively low and it can take weeks to obtain sufficient cells for downstream experiments. In chip cultures, the number of cells required limited our experimental set-up and sometimes necessitates the use of donor mixes. Possibly by tuning for example FGFR2-mediated signaling via additions to the medium, a more proliferative phenotype can be promoted (29). Another limitation is that we could not identify a dominant factor that was predictive to the success rate of initial organoid formation or propagation. The success of AEC2 organoid culture initiation and expansion was highly donor-dependent but did not seem related to the size of the tissue or the number of cells the culture was started with. Furthermore, the interdonor variation with respect to the number of days required before first passaging was large and was also not related to the number of cells isolated. Further studies and larger donor numbers are needed to identify if and which donor characteristics determine the success of organoid initiation and AEC2 expansion.

In conclusion, we demonstrate the successful isolation of AEC2s from (diseased) human lung tissue that can subsequently be expanded using feeder-free organoid culture. The organoids retain expression of AEC2 markers over time and can be dissociated for further experiments. We furthermore showed the feasibility of culturing these patient-derived AEC2s in the chip under application of cyclic strain. This method is expected to aid future research into the way forces related to breathing affect the alveolar compartment, which is needed in view of the important gaps in our knowledge on the cellular biomechanics of the human alveolus in health and disease.

## DATA AVAILABILITY

Data will be made available on reasonable request.

## SUPPLEMENTAL DATA

10.6084/m9.figshare.17125061 Supplemental Figs. S1 and S2: https://doi.org/10.6084/m9.figshare.17125061.

## GRANTS

This study was supported by a grant of the Lung Foundation Netherlands (Grant No. 6.1.14.010), The Netherlands Organization for Health Research and Development (ZonMw; Grant No. 114021508), Translationeel Adult Stamcelonderzoek—ZonMw (TAS-ZonMW; Grant No. 40414009816007), EU Marie Curie Global Fellowship (Grant No. 748569), and Stichting Proefdiervrij.

## DISCLOSURES

Anne M. van der Does was supported by a Global Marie Curie fellowship (No. 748569) that included a 1-yr visit at Emulate Inc. to work on their Lung-Chips. Materials from Emulate related to this work were therefore provided by Emulate. None of the other authors has any conflicts of interest, financial or otherwise, to disclose.

## AUTHOR CONTRIBUTIONS

S.v.R., A.v.S., P.P.S.J.K., P.S.H., and A.M.v.d.D. conceived and designed research; S.v.R., A.v.S., P.P.S.J.K., R.W.A.L.L., M.B., and A.M.v.d.D. performed experiments; S.v.R., A.v.S., P.P.S.J.K., R.W.A.L.L., M.B., and A.M.v.d.D. analyzed data; S.v.R., A.v.S., P.P.S.J.K., R.W.A.L.L., M.B., J.S., P.S.H., and A.M.v.d.D. interpreted results of experiments; S.v.R., A.v.S., R.W.A.L.L., M.B. and J.S. prepared figures; S.v.R. and A.M.v.d.D. drafted manuscript; S.v.R., A.v.S., P.P.S.J.K., M.B., J.S., P.S.H., and A.M.v.d.D. edited and revised manuscript; S.v.R., A.v.S., P.P.S.J.K., R.W.A.L.L., M.B., J.S., P.S.H., and A.M.v.d.D. approved final version of manuscript.

## References

[1] World Health Organization. The top 10 causes of death (Online). https://www.who.int/news-room/fact-sheets/detail/the-top-10-causes-of-death [Dec 9, 2020].

[2] Khakban A, Sin DD, FitzGerald JM, McManus BM, Ng R, Hollander Z, Sadatsafavi M. The projected epidemic of chronic obstructive pulmonary disease hospitalizations over the next 15 years. a population-based perspective. Am J Respir Crit Care Med 195: 287–291, 2017. doi:10.1164/rccm.201606-1162PP. 27626508

[3] Hogg JC, Timens W. The pathology of chronic obstructive pulmonary disease. Annu Rev Pathol 4: 435–459, 2009. doi:10.1146/annurev.pathol.4.110807.092145. 18954287

[4] Agustí A, Hogg JC. Update on the pathogenesis of chronic obstructive pulmonary disease. N Engl J Med 381: 1248–1256, 2019. doi:10.1056/NEJMra1900475. 31553836

[5] Miravitlles M, Vogelmeier C, Roche N, Halpin D, Cardoso J, Chuchalin AG, Kankaanranta H, Sandström T, Śliwiński P, Zatloukal J, Blasi F. A review of national guidelines for management of COPD in Europe. Eur Respir J 47: 625–637, 2016. doi:10.1183/13993003.01170-2015. 26797035 PMC4733567

[6] Celli BR, Wedzicha JA. Update on clinical aspects of chronic obstructive pulmonary disease. N Engl J Med 381: 1257–1266, 2019. doi:10.1056/NEJMra1900500. 31553837

[7] Uhl EW, Warner NJ. Mouse models as predictors of human responses: evolutionary medicine. Curr Pathobiol Rep 3: 219–223, 2015. doi:10.1007/s40139-015-0086-y. 26246962 PMC4522464

[8] Jacob A, Morley M, Hawkins F, McCauley KB, Jean JC, Heins H, Na CL, Weaver TE, Vedaie M, Hurley K, Hinds A, Russo SJ, Kook S, Zacharias W, Ochs M, Traber K, Quinton LJ, Crane A, Davis BR, White FV, Wambach J, Whitsett JA, Cole FS, Morrisey EE, Guttentag SH, Beers MF, Kotton DN. Differentiation of human pluripotent stem cells into functional lung alveolar epithelial cells. Cell Stem Cell 21: 472–488.e410, 2017. doi:10.1016/j.stem.2017.08.014. 28965766 PMC5755620

[9] Witherden IR, Tetley TD. Isolation and culture of human alveolar type II pneumocytes. Methods Mol Med 56: 137–146, 2001. doi:10.1385/1-59259-151-5:137. 21336897

[10] Hiemstra PS, Tetley TD, Janes SM. Airway and alveolar epithelial cells in culture. Eur Respir J 54: 1900742, 2019. doi:10.1183/13993003.00742-2019. 31515398

[11] Mao P, Wu S, Li J, Fu W, He W, Liu X, Slutsky AS, Zhang H, Li Y. Human alveolar epithelial type II cells in primary culture. Physiol Rep 3: e12288, 2015. doi:10.14814/phy2.12288. 25677546 PMC4393197

[12] Glisinski KM, Schlobohm AJ, Paramore SV, Birukova A, Moseley MA, Foster MW, Barkauskas CE. Interleukin-13 disrupts type 2 pneumocyte stem cell activity. JCI Insight 5: e131232, 2020. doi:10.1172/jci.insight.131232. 31941839 PMC7030850

[13] Salahudeen AA, Choi SS, Rustagi A, Zhu J, van Unen V, de la O SM, Flynn RA, Margalef-Català M, Santos AJM, Ju J, Batish A, Usui T, Zheng GXY, Edwards CE, Wagar LE, Luca V, Anchang B, Nagendran M, Nguyen K, Hart DJ, Terry JM, Belgrader P, Ziraldo SB, Mikkelsen TS, Harbury PB, Glenn JS, Garcia KC, Davis MM, Baric RS, Sabatti C, Amieva MR, Blish CA, Desai TJ, Kuo CJ. Progenitor identification and SARS-CoV-2 infection in human distal lung organoids. Nature 588: 670–675, 2020. doi:10.1038/s41586-020-3014-1. 33238290 PMC8003326

[14] Katsura H, Sontake V, Tata A, Kobayashi Y, Edwards CE, Heaton BE, Konkimalla A, Asakura T, Mikami Y, Fritch EJ, Lee PJ, Heaton NS, Boucher RC, Randell SH, Baric RS, Tata PR. Human lung stem cell-based alveolospheres provide insights into SARS-CoV-2-mediated interferon responses and pneumocyte dysfunction. Cell Stem Cell 27: 890–904.e8, 2020. doi:10.1016/j.stem.2020.10.005. 33128895 PMC7577733

[15] Evans KV, Lee JH. Alveolar wars: the rise of in vitro models to understand human lung alveolar maintenance, regeneration, and disease. Stem Cells Transl Med 9: 867–881, 2020. doi:10.1002/sctm.19-0433. 32272001 PMC7381809

[16] Benam KH, Villenave R, Lucchesi C, Varone A, Hubeau C, Lee HH, Alves SE, Salmon M, Ferrante TC, Weaver JC, Bahinski A, Hamilton GA, Ingber DE. Small airway-on-a-chip enables analysis of human lung inflammation and drug responses in vitro. Nat Methods 13: 151–157, 2016. doi:10.1038/nmeth.3697. 26689262

[17] Zamprogno P, Wüthrich S, Achenbach S, Thoma G, Stucki JD, Hobi N, Schneider-Daum N, Lehr CM, Huwer H, Geiser T, Schmid RA, Guenat OT. Second-generation lung-on-a-chip with an array of stretchable alveoli made with a biological membrane. Commun Biol 4: 168, 2021. doi:10.1038/s42003-021-01695-0. 33547387 PMC7864995

[18] Huh D, Matthews BD, Mammoto A, Montoya-Zavala M, Hsin HY, Ingber DE. Reconstituting organ-level lung functions on a chip. Science 328: 1662–1668, 2010. doi:10.1126/science.1188302. 20576885 PMC8335790

[19] Schrumpf JA, Ninaber DK, van der Does AM, Hiemstra PS. TGF-β1 impairs vitamin d-induced and constitutive airway epithelial host defense mechanisms. J Innate Immun 12: 74–89, 2020. doi:10.1159/000497415. 30970352 PMC6959102

[20] Sachs N, Papaspyropoulos A, Zomer-van Ommen DD, Heo I, Bottinger L, Klay D, et al. Long-term expanding human airway organoids for disease modeling. EMBO J 38: e100300, 2019. doi:10.15252/embj.2018100300. 30643021 PMC6376275

[21] Gonzalez RF, Allen L, Gonzales L, Ballard PL, Dobbs LG. HTII-280, a biomarker specific to the apical plasma membrane of human lung alveolar type II cells. J Histochem Cytochem 58: 891–901, 2010. doi:10.1369/jhc.2010.956433. 20566753 PMC2942742

[22] Hassell BA, Goyal G, Lee E, Sontheimer-Phelps A, Levy O, Chen CS, Ingber DE. Human organ chip models recapitulate orthotopic lung cancer growth, therapeutic responses, and tumor dormancy in vitro. Cell Rep 21: 508–516, 2017 [Erratum in *Cell Rep* 23: 3698, 2018]. doi:10.1016/j.celrep.2017.09.043. 29020635

[23] Huh DD. A human breathing lung-on-a-chip. Ann Am Thorac Soc 12, Suppl 1: S42–S44, 2015. doi:10.1513/AnnalsATS.201410-442MG. 25830834 PMC5467107

[24] Waters CM, Roan E, Navajas D. Mechanobiology in lung epithelial cells: measurements, perturbations, and responses. Compr Physiol 2: 1–29, 2012. doi:10.1002/cphy.c100090. 23728969 PMC4457445

[25] Fredberg JJ, Inouye D, Miller B, Nathan M, Jafari S, Raboudi SH, Butler JP, Shore SA. Airway smooth muscle, tidal stretches, and dynamically determined contractile states. Am J Respir Crit Care Med 156: 1752–1759, 1997. doi:10.1164/ajrccm.156.6.9611016. 9412551

[26] Tsutsumi A, Ozaki M, Chubachi S, Irie H, Sato M, Kameyama N, Sasaki M, Ishii M, Hegab AE, Betsuyaku T, Fukunaga K. Exposure to cigarette smoke enhances the stemness of alveolar type 2 cells. Am J Respir Cell Mol Biol 63: 293–305, 2020. doi:10.1165/rcmb.2019-0188OC. 32338993

[27] Lamers MM, van der Vaart J, Knoops K, Riesebosch S, Breugem TI, Mykytyn AZ, Beumer J, Schipper D, Bezstarosti K, Koopman CD, Groen N, Ravelli RBG, Duimel HQ, Demmers JAA, Verjans G, Koopmans MPG, Muraro MJ, Peters PJ, Clevers H, Haagmans BL. An organoid-derived bronchioalveolar model for SARS-CoV-2 infection of human alveolar type II-like cells. EMBO J 40: e105912, 2021. doi:10.15252/embj.2020105912. 33283287 PMC7883112

[28] Dietl P, Haller T. Exocytosis of lung surfactant: from the secretory vesicle to the air-liquid interface. Annu Rev Physiol 67: 595–621, 2005. doi:10.1146/annurev.physiol.67.040403.102553. 15709972

[29] Liberti DC, Kremp MM, Liberti WA 3rd, Penkala IJ, Li S, Zhou S, Morrisey EE. Alveolar epithelial cell fate is maintained in a spatially restricted manner to promote lung regeneration after acute injury. Cell Rep 35: 109092, 2021. doi:10.1016/j.celrep.2021.109092. 33979629 PMC8220578

[30] Ravasio A, Olmeda B, Bertocchi C, Haller T, Pérez-Gil J. Lamellar bodies form solid three-dimensional films at the respiratory air-liquid interface. J Biol Chem 285: 28174–28182, 2010. doi:10.1074/jbc.M110.106518. 20558742 PMC2934682

[31] Kobayashi Y, Tata A, Konkimalla A, Katsura H, Lee RF, Ou J, Banovich NE, Kropski JA, Tata PR. Persistence of a regeneration-associated, transitional alveolar epithelial cell state in pulmonary fibrosis. Nat Cell Biol 22: 934–946, 2020. doi:10.1038/s41556-020-0542-8. 32661339 PMC7461628

[32] Nabhan AN, Brownfield DG, Harbury PB, Krasnow MA, Desai TJ. Single-cell Wnt signaling niches maintain stemness of alveolar type 2 cells. Science 359: 1118–1123, 2018. doi:10.1126/science.aam6603. 29420258 PMC5997265

[33] Zhao L, Yee M, O'Reilly MA. Transdifferentiation of alveolar epithelial type II to type I cells is controlled by opposing TGF-β and BMP signaling. Am J Physiol Lung Cell Mol Physiol 305: L409–L418, 2013. doi:10.1152/ajplung.00032.2013. 23831617 PMC3763037

[34] Choi J, Park JE, Tsagkogeorga G, Yanagita M, Koo BK, Han N, Lee JH. Inflammatory signals induce AT2 cell-derived damage-associated transient progenitors that mediate alveolar regeneration. Cell Stem Cell 27: 366–382.e7, 2020. doi:10.1016/j.stem.2020.06.020. 32750316 PMC7487779

[35] Park JS, Burckhardt CJ, Lazcano R, Solis LM, Isogai T, Li L, Chen CS, Gao B, Minna JD, Bachoo R, DeBerardinis RJ, Danuser G. Mechanical regulation of glycolysis via cytoskeleton architecture. Nature 578: 621–626, 2020. doi:10.1038/s41586-020-1998-1. 32051585 PMC7210009

